# Region-Based PDEs for Cells Counting and Segmentation in 3D+Time Images of Vertebrate Early Embryogenesis

**DOI:** 10.1155/2009/968986

**Published:** 2009-10-08

**Authors:** Barbara Rizzi, Alessandro Sarti

**Affiliations:** ^1^DEIS, University of Bologna, Via Venezia 52, 47521 Cesena, Italy; ^2^DEIS, University of Bologna, Via Risorgimento 2, 40136 Bologna, Italy

## Abstract

This paper is devoted to the segmentation of cell nuclei from time lapse confocal microscopy images, taken throughout early Zebrafish embryogenesis. The segmentation allows to identify and quantify the number of cells in the animal model. This kind of information is relevant to estimate important biological parameters such as the cell proliferation rate in time and space. Our approach is based on the active contour model without edges. We compare two different formulations of the model equation and evaluate their performances in segmenting nuclei of different shapes and sizes. Qualitative and quantitative comparisons are performed on both synthetic and real data, by means of suitable gold standard. The best approach is then applied on a number of time lapses for the segmentation and counting of cells during the development of a zebrafish embryo between the sphere and the shield stage.

## 1. Introduction

The cell morphodynamics underlying the processes that control the organized spatial distribution of cells during the embryonic development are nowadays largely unknown. A single fertilized egg-cell (zygote) undergoes cellular divisions, differentiations, and interactions giving rise to specific forms of tissues and organs. The reconstruction of the nuclei shape and the identification of their number and position during embryogenesis are an essential tool for an integrated understanding of such morphogenetic processes. This kind of information is relevant to estimate the cell growth and identify cell divisions and apoptosis. It is a helpful tool to extract parameters such as the cell proliferation rate in time and space. It is relevant for investigating stem cells populations and early steps of cancerogenesis, opening the way for the preclinical evaluation of anticancer drug effects in vivo. We apply in this paper a region-based segmentation algorithm to segment the nuclei in a live zebrafish embryo. Since during the acquisition the embryo is growing up, the cell number strongly increases. The segmentation easily allows to identify the nuclei and count their number in time. Furthermore, since prior and during mitosis the nuclei condense and lengthen along the future cell division plane, the segmentation is a helpful tool to identify cell divisions. Our approach is based on the active contour model without edges. We consider and compare two different forms of the equation originally proposed by Chan and Vese [1]. Comparison is performed through qualitative and quantitative analysis of results on both phantoms and real data. We then apply to our dataset the formulation that ensures the best performances.

In the rest of the paper we first describe the data acquisition process (Section 2). In Section 3 we introduce the active contour model without edges and both tested formulations. In Section 4 we show and quantify results and justify our choice of method and parameters. Conclusions are provided in Section 5.

## 2. Image Acquisition

### 2.1. The Animal Model

The zebrafish (Danio Rerio) is a model organism extensively studied to explore the gene function and vertebrate development [2–7] and might soon become a major model organism for preclinical drug testing by pharmaceutical industries. Although distant from humans, nevertheless it has comparable organs and tissues and may supplement higher vertebrate models. Compared with mice, zebrafish embryo develops rapidly and externally to the mother, favoring its manipulation. Its transparency allows the visual inspection of the internal anatomy. Due to its ability to regenerate fins, skin, the heart, and the brain, it is an object of study to understand healing/repair mechanisms in vertebrates.

### 2.2. Animal Preparation and Laser Confocal Microscopy Set-Up

To reconstruct the shape and the position of every nucleus in a live zebrafish embryo is necessary to stain all the cells and use an acquisition technique with micrometrical resolution. This is a very challenging approach requiring specific methodologies and tools in embryos engineering and microscopy imaging. Recent advances in imaging strategies open the way to in 4D imaging of live animals with a resolution at the cellular level and enough contrast to allow segmentation of individual cells. Through the ubiquitous expression of fluorescent proteins in the zebrafish embryo, it is possible to label all the cells and perform time-lapse CLSM (confocal laser scanning microscopy) imaging throughout embryonic development.

**Figure 1:**
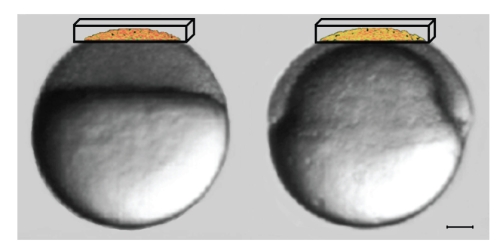
Zebrafish embryo. Scalar bar is 100 *μ*m. The imaged volume is represented in volume rendering inside the black box. Left: embryo at 3.5 hours post fertilization (sphere stage). Right: embryo at 7.5-hour postfertilization (shield stage).

The embryo used in this paper to design and validate our strategy has been stained through injection at the one cell stage of RNAs encoding farnesylated mCherry fluorescent protein and histone H2B/eGFP fusion protein. This allows the simultaneous acquisition of two different signals, membranes and nuclei, respectively. In this paper we focus our attention on nuclei. The images have been acquired using a Confocal Microscope Leica SP2 AOBS with a 40x/0.8 NA water objective and 488 nm and 561 nm laser light excitation. We used the best compromise in terms of embryo survival and spatial and temporal resolution, as suggested in [8]. The data has been acquired for four hours (25°C under the microscope), starting at 3.5-hour postfertilization (development at 28°C) from the animal pole. Due to the lower temperature, during the acquisition the normal embryo development [9] is slowed down and by the end it reaches the shield stage (see Figure 1).

Images are acquired parallel to the focal plane *xy*, at regularly spaced *z* depth to build a *z* stack. The procedure has been repeated every 5 minutes to generate a temporal sequence of 49 volumes. Every volume consists of 512 × 512 × 30 voxels. The dimension of each voxel is 0.58 × 0.58 × 1.04 *μ* ^3^. In the entire period of development, the whole embryo looks like a sphere with a diameter of 740 *μ*. As the 3D images have a physical dimension of 300 × 300 × 31 *μ* ^3^, they cover only the top part of the embryo, as depicted in Figure 1.

**Figure 2:**
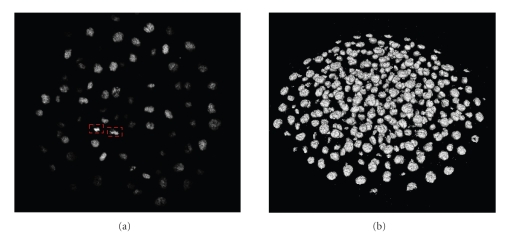
Original data. Volume selected in the middle of the time lapse series. (a) Orthoslice on the *xy* plane. Nuclei in mitosis are surrounded by a red rectangle in dotted line. (b) Isosurface value of 30.

Observing Figure 2(a) is possible to note how the typology of staining influences the features of original data. As the histone H2B protein is a component of the chromatin, nuclei in mitosis appear well contrasted, as chromosomes are condensing along the future division plane. Conversely cell nuclei in interphase show a nonuniform image intensity and boundaries often not clearly defined. We explain in next section how this peculiarity leads to the choice of the segmentation method.

## 3. Image Segmentation

One of the most popular approaches for image segmentation is the active contour model or snake [10], based on the evolution of a curve attracted by image boundaries in order to detect objects. Malladi et al. [11] and Caselles et al. [12] implemented such models using level set methods, introduced first by Osher and Sethian [13] and extensively used to track the evolution of fronts in a variety of applications. With the level set methods the desired curve of active contour models is embedded as zero level set of an implicit function, overcoming the drawbacks of the original snakes models [10] as the difficulties related to topological transformations. However, the above-mentioned active contour models are edge-based; that is, a curve is evolved with a speed function depending on a precomputed edge indicator defined by the image gradient. Dealing with image derivatives, a preprocessing step is required to remove the noise and smooth the image. If the objects are not well contrasted, the denoising can disrupt image information and preclude the correct identification of objects surface. Lately a region-based level-set method has been proposed by Chan and Vese [1] to segment objects whose boundaries are not defined by a gradient. The model does not require preprocessing and is based on the informations provided directly by the image intensity. The Chan Vese approach is then well adapted to situations in which images are noisy, and it is difficult to extract the boundary of the desired object. These features make the method well suitable to our typology of data.

### 3.1. The Chan Vese Model

Let us consider a 3D image *I* as a real positive function defined in a rectangular domain Ω ∈ ℝ^3^. The Chan Vese [1] model moves an arbitrary surface *S* ∈ Ω for decomposing the image *I* into two regions of approximately piecewise-constant intensities: the objects to be detected and the background. Such decomposition is obtained by minimizing the following energy functional:

(1) $$\begin{array}{ll} E\left(S,s_{i},s_{o}\right) & =\mu \cdot \left(\text{area}\left(S\right)\right)\\ & \;+\lambda_{i}\int_{\text{inside}(S)}{\left|I(x,y,z)-s_{i}\right|}^{2}dx\;dy\;dz\\ & \;+\lambda_{o}\int_{\text{outside}(S)}{\left|I(x,y,z)-s_{o}\right|}^{2}dx\;dy\;dz, \end{array}$$

where *s*_*i*_ and *s*_*o*_ are, respectively, the averages of *I* inside and outside *S*, and *μ* ≥ 0, *λ*_*i*_ > 0, and *λ*_*o*_ > 0 are fixed parameters. While the first term of the right side of (1) controls its smoothness, the surface *S* separates the image into distinct regions depending on their mean values. The parameter *μ* plays an important role in the segmentation process, as it acts like a scale parameter. The minimization problem is solved using the level set method. Let *ω* ⊂ Ω, *S* is represented as a zero level set of an implicit function *ϕ*: Ω → ℝ such that

(2) $$\begin{array}{ll} & S=\partial \omega =\left\{\left(x,y,z\right)\in \Omega :\phi \left(x,y,z\right)=0\right\},\\ & \text{inside}\left(S\right)=\omega =\left\{\left(x,y,z\right)\in \Omega :\phi \left(x,y,z\right)>0\right\},\\ & \text{outside}\left(S\right)=\Omega \setminus \overset{̅}{\omega }=\left\{\left(x,y,z\right)\in \Omega :\phi \left(x,y,z\right)<0\right\}. \end{array}$$

The level set flow associated to (1) is computed as

(3) $$\begin{array}{ll} & \phi_{t}=\delta \left(\phi \right)\left(\mu \nabla \cdot \frac{\nabla \phi }{\left|\nabla \phi \right|}-\lambda_{i}{\left(I-s_{i}\right)}^{2}+\lambda_{o}{\left(I-s_{o}\right)}^{2}\right),\\ & \text{in}\;\;\Omega \times ]0,\infty [,\\ & \phi \left(x,y,z,0\right)=\phi_{0}\left(x,y,z\right)\;\text{in}\;\;\Omega ,\\ & \frac{\delta \left(\phi \right)}{\left|\nabla \phi \right|}\frac{\partial \phi }{\partial n}=0\;\text{in}\;\;\partial \Omega , \end{array}$$

where *δ*(·) is the Dirac function, *n* denotes the exterior normal to the boundary ∂Ω, and *ϕ*_0_(*x*, *y*, *z*) is an initial function with the property that its zero level set corresponds to the position of the initial front. For details about using the level set method to solve (1) we refer to the original paper [1].

### 3.2. Replacement of the *δ* Function

Equation (3) acts only locally, on the zero level set of the function *ϕ*, and the solution depends on the initial condition. In order to extend the evolution to all the level sets of *ϕ* and find a global minimizer of the energy, a common approach [14, 15] is to set *δ*(*ϕ*) = |∇*ϕ*|, leading to

(4) $$\phi_{t}=\left|\nabla \phi \right|\left(\mu K-\lambda_{i}{\left(I-s_{i}\right)}^{2}+\lambda_{o}{\left(I-s_{o}\right)}^{2}\right),$$

where *K* = ∇ · (∇(*ϕ*)/|∇(*ϕ*)|) is the mean curvature of every level sets of *ϕ*. The term *K*|∇(*ϕ*)| performs then a regularization through a mean curvature motion.

Another interesting solution has been proposed by Gibou and Fedkiw [16] who take *δ* = 1 (see also [17–19]). Observing that only the zero level set of *ϕ* is important and the object boundaries are represented by discontinuities in *ϕ*, they ignore the regularization term and consider directly the ordinary differential equation *ϕ*_*t*_ = −*λ*_*i*_(*I* − *s*_*i*_)^2^ + *λ*_*o*_(*I* − *s*_*o*_)^2^. Regularization and scaling are achieved by a preprocessing step of nonlinear diffusion [20].

Considering the features of dataset, we suggest instead, following [21], to avoid any denoising and take *δ* = 1 keeping the regularization term in (3):

(5) $$\phi_{t}=\mu K-\lambda_{i}{\left(I-s_{i}\right)}^{2}+\lambda_{o}{\left(I-s_{o}\right)}^{2}.$$

The first term in (5) represents then a total variation regularization [22]. Its use in the Chan Vese model has been already proposed in [23].

We tested the behavior of (5) and (4) on noisy phantoms and our real data. To solve the presented PDEs models we used explicit finite difference schemes as introduced in [13]. In the next section we show qualitative and quantitative results justifying our choice of *δ* representation.

## 4. Results

As previously introduced in Section 2.2, our dataset is composed by 49 volumes and represents the cell nuclei during the development of a zebrafish embryo between the sphere and the shield stage. To a first approximation (see Figure 2) the nuclei can be comparable to spheres of different diameter, between 6-7 (internal) and 12–14 voxels (epithelial). Nevertheless, sometimes they are elongated and very jagged, especially before a mitosis. Furthermore, the nuclei located in depth are usually incomplete. As our aim is to segment all the nuclei at the same time, we have to segment simultaneously objects of different shapes and sizes. In the next section we compare from this perspective the behavior of both versions of Chan-Vese model previously introduced. We evaluate their performances according to different values of scale parameter *μ* on a synthetic image and our real data.

### 4.1. Choice of Method and Tuning of *μ*

The parameter *μ* determines an upper bound to the mean curvature of segmented objects. Therefore if we have to detect many objects with different size, *μ* should be small. If we have to detect only larger objects and to filter out smaller objects (like points, due to the noise), then *μ* has to be larger. By considering the nuclei dimension, we perform a first test on phantoms by segmenting spheres of different radii, then on real data by segmenting a region of interest selected in a volume of time lapse series. We test both (4) and (5) using different values of *μ*. In all our computations we use *λ*_*i*_ = *λ*_*o*_ = 1. The time step is set to 0.1 if *μ* ≤ 0.5, otherwise to 0.01. Equations (4) and (5) are iterated until the steady state. The segmentation error is evaluated by calculating the mean Hausdorff distance (MHD) and the Hausdorff distance (HD) [24] between the segmented surfaces and a suitable gold standard. Given two finite point sets, *A* = {*a*_1_,…, *a*_*p*_} and *B* = {*b*_1_,…, *b*_*q*_}, the Hausdorff distance is defined as

(6) HD(A,B)=max(h(A,B),h(B,A)),

where *h*(*A*, *B*) = max_*a*∈*A*_ min_*b*∈*B*_ ‖*a* − *b*‖.

**Figure 3:**
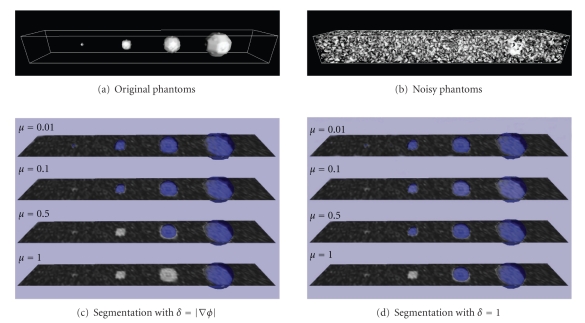
Graphical representation of the calibration of *μ*. Noisy spheres of different radii (1, 3, 5, and 7 voxels) segmented using different values of scale parameter. Figures (c) and (d) show the noisy data in a cutting plane and the segmentation (blue) in surface rendering. (a) Original spheres. (b) Noisy spheres (Gaussian noise with mean value 0 and standard deviation 50). (c) Segmentation with *δ* = |∇*ϕ*|. (d) Segmentation with *δ* = 1. Steady state after 1000 iterations for *μ* ≤ 0.1, otherwise 7000 (a) and 9000 (b) iterations.

**Figure 4:**
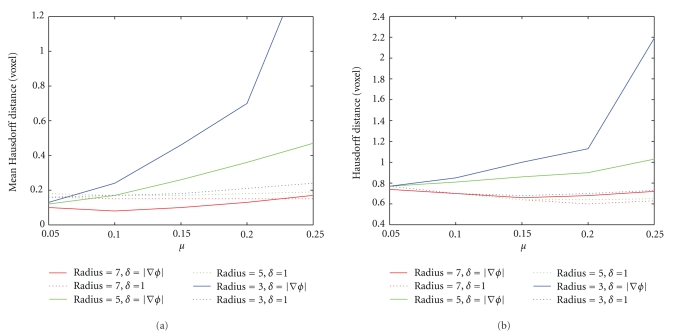
Graph of Mean Hausdorff Distance and Hausdorff Distance between surfaces of original and segmented spheres. The distances are expressed in function of scale parameter. Solid line represents results using (4). Dashed line represents results using (5) are in red, spheres of radius 7. 5 in green, and 3 in blue. The model described in (5) is more robust than the model given by (4).

**Figure 5:**
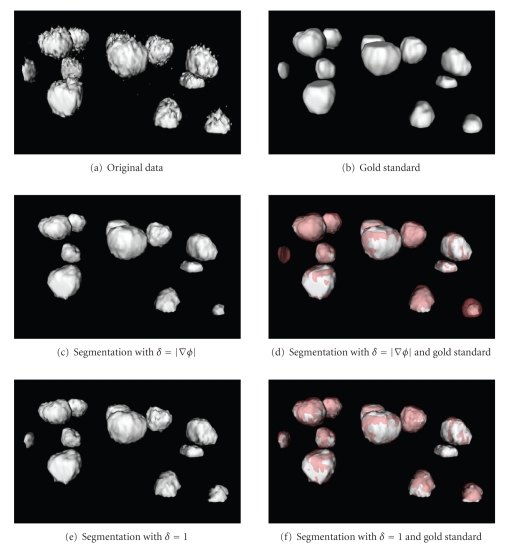
A region of interest selected on real data, gold standard obtained by manual segmentation, and surfaces segmented using (4) and (5). In every case *μ* = 0.05. Steady state is, respectively, after 1300 and 1500 iterations. (a) Isosurface of original data. (b) Gold standard. (c) Segmentation with *δ* = |∇*ϕ*|. (d) Segmentation with *δ* = |∇*ϕ*| superimposed to gold standard (red, transparent). (c) Segmentation with *δ* = 1. (d) Segmentation with *δ* = 1 superimposed to gold standard (red, transparent). Using *δ* = |∇*ϕ*| is not possible to segment all the nuclei in the region.

The MHD is defined similarly, using *h*(*A*, *B*) = 1/*p*∑_*i* = 1_^*p*^min_*b*∈*B*_ ‖*a*_*i*_ − *b*‖. The two formulations can be used to quantify different kinds of error: HD is related to the maximum segmentation error, whereas MHD to the total error. In Figure 3 are depicted our results on noisy phantoms, using, respectively, (Figures 3(c) and 3(d)) (4) and (5). The original data (Figures 3(a), 3(b)) are composed of spheres with radius 1, 3, 5, and 7 voxels. Figures 3(c) and 3(d)  show the original data in a cutting plane and the segmentation (blue) obtained using *μ* = 0.01, 0.1, 0.5,1. Considering the nuclei size in our dataset, our purpose is to segment only spheres with radius ≥3. We can first observe how increasing *μ* only bigger objects can be detected with both methods. When *μ* = 0.01, (4) is able to identify all the four spheres, while (5) segments also smaller regions given by noise. For *μ* = 0.1 both methods segment correctly the desired regions. When the scale parameter is 0.5, only (5) still segments three regions. For *μ* = 1, (4) identifies one sphere and (5) two. We verified that (4) is able to detect three regions only when 0.05 ≤ *μ* ≤ 0.25, while (5) can exploit a more wide range, 0.05 ≤ *μ* ≤ 0.80. Such behavior can be observed also by examining the mean and the maximum segmentation errors. The graphs in Figure 4 depict MHD and HD calculated between the surfaces of segmented spheres and corresponding surfaces of spheres created without noise. The results are plotted only for the range of interest of *μ*. In solid line is the use of (4). In dashed line is the use of (5). The color is related to the radius. The sphere of the biggest radius (7 voxels, depicted in red) is segmented correctly with both methods and for the whole range of scale parameter. The behavior of methods changes instead for smaller spheres. While using (5) errors keep about constant for all nuclei; errors with (4) are everywhere small only for the smallest value of *μ*. The replacement of *δ* with 1 in (3) is less sensitive to changes in size than its replacement with |∇*ϕ*|. Such behavior is confirmed on real data, also if in a more restrictive useful range of values for *μ*. We selected a region of interest in a volume of our dataset. The ROI contains both epithelial and internal nuclei, and some of them are cut. A gold standard has been created by manual segmentation to compare the methods. Original data, gold standard, and results attained using *μ* = 0.05 are depicted in Figure 5. For smaller values of *μ* both methods detect regions given by noise. We can observe that the methods described in (4) fails, because it is not able to segment all the nuclei in the subvolume. The smallest region (on the left in Figures 5(c)  and 5(d)) is lost. Another small nucleus (bottom-right in the same figures) is not correctly segmented. The method described by (5) is instead able to segment all the regions in the ROI. Its mean and maximum errors are listed in Table 1. The Hausdorff and the mean Hausdorff Distances are in this case averaged on the number of nuclei in the subvolume. As the spacing in our data is not uniform, distances are given in *μ*m. For *μ* = 0.05, 0.1 the total error is small (≈0.3 *μ*m) and the maximum is anyhow limited (≈1.5 *μ*m). The diagonal of every voxel is in fact about 1.2 *μ*m.

Because of robustness, we then choose to segment our time lapse series with the method described by (4). Considering results on both phantoms and real data, we used *μ* = 0.1. Let us point out that this analysis represents also a validation of segmentation, with very satisfactory results.

### 4.2. Segmentation and Cells Counting

We show in this section some results obtained by segmenting the whole time lapse with the method described by (5). The level-set function is initialized as the difference between the 3D image *I* itself and its mean value. The mean value is calculated only where *I* > 0 and the function thus constructed is then smoothed with few scale steps of the heat equation. This initialization provides an initial solution close to the final one and does not depend on a previous segmentation. Actually an alternative could be to use the result of segmentation at time *t* as initial solution for the segmentation at time *t* + 1. But this choice does not consider that the number of nuclei changes between subsequent volumes. Indeed some nuclei divide and others are close to the bottom of the acquired volume and can go out of the imaged part of the embryo. Moreover, our solution allows the simultaneous segmentation, for example, on different processors, of all the volumes in the time lapse series.

We keep *λ*_*i*_ = *λ*_*o*_ = 1, while the scale parameters have been chosen by our previous analysis and set to 0.1. We use in every case a time step of 0.1 and the steady state is reached after about 1500 iterations. From the isosurface corresponding to the zero level set of the final solution are then extracted the connected components, whose number provides the counting of nuclei in the processed volume. Both for extracting the isosurface zero and its connected components we make use of the open-source VTK (Visualization Toolkit) library [25], which provides widely used filters for image visualization and analysis.

**Figure 6:**
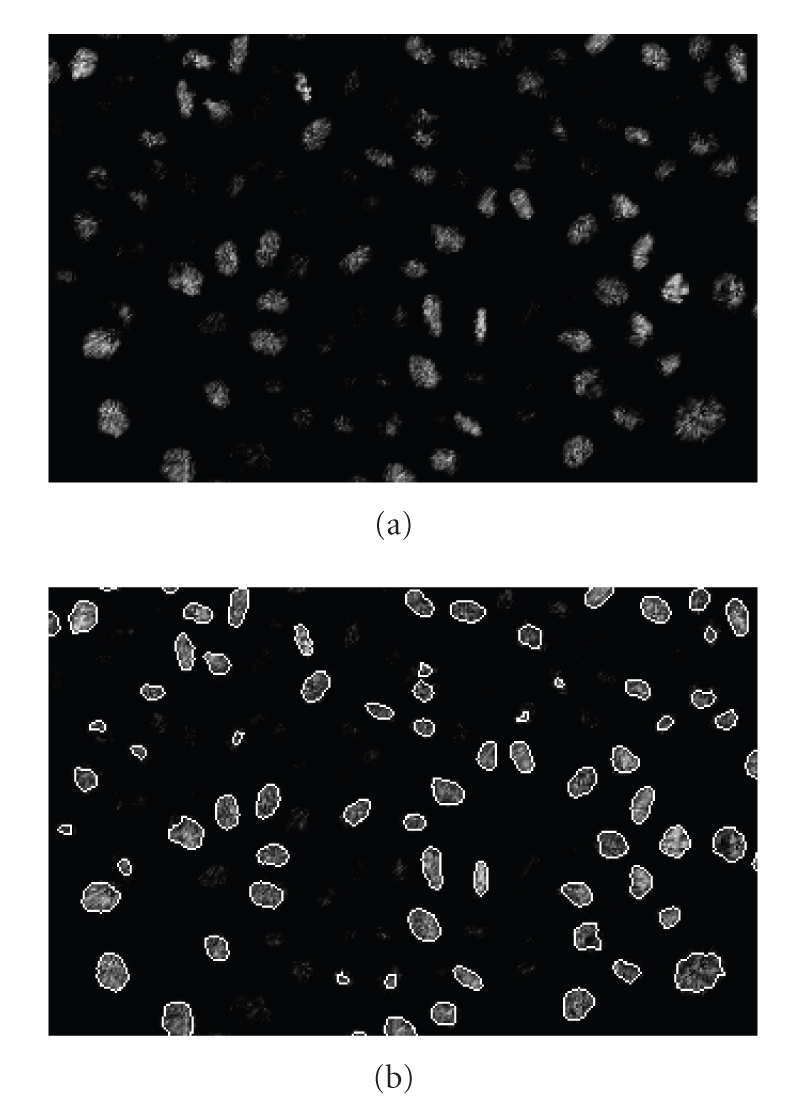
(a) A slice cut of the original data and (b) their overlapping with a cut of the segmented surfaces.

**Figure 7:**
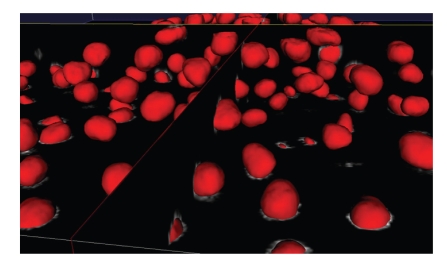
Isosurface of nuclei segmentation superimposed to three orthoslices of raw data.

**Figure 8:**
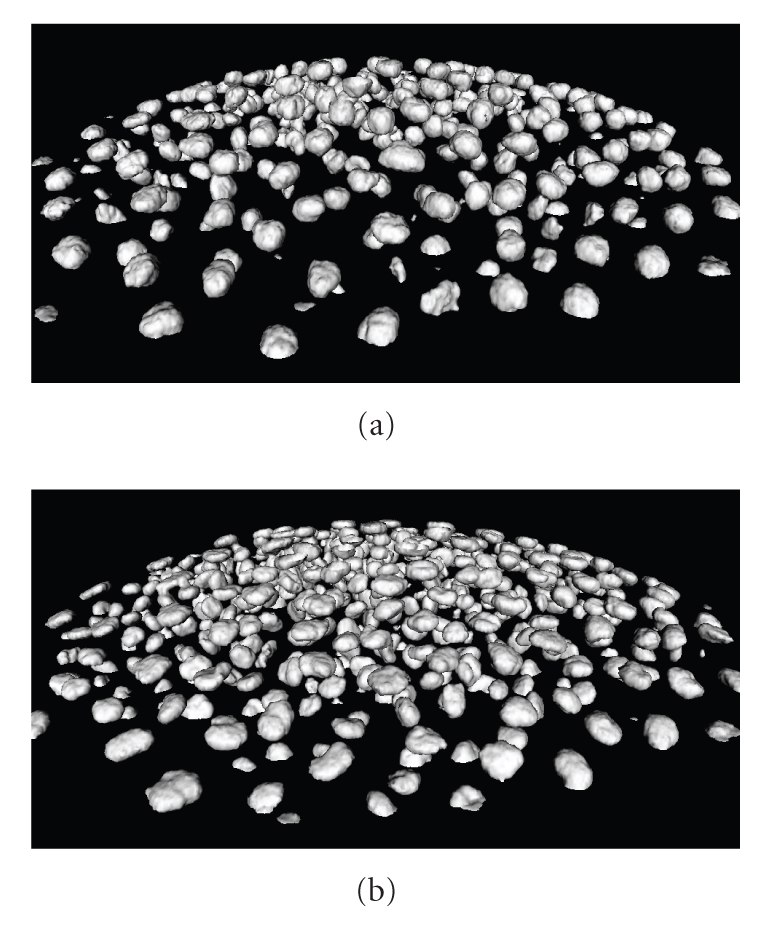
Segmentation of first and last volume of time lapse series.

**Figure 9:**
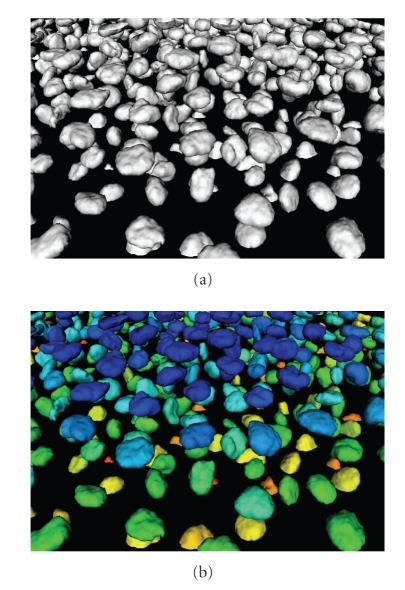
Segmentation of last volume of time lapse series. (a) Segmentation. (b) Connected components.

**Figure 10:**
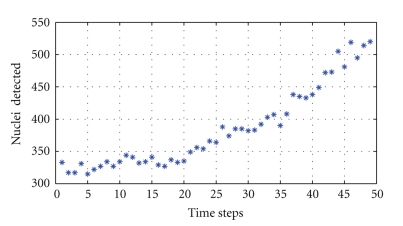
Number of nuclei detected in the whole time lapse series.

Examples of our segmentations on whole 3D images are given in Figures 6–8. In the first a cutting plane of last volume is compared with a cut of segmented surfaces. The white outline well reproduces the nuclei boundaries even if they are not clearly defined, and small regions are correctly detected. In Figure 7 is visualized a segmentation (red) together with three orthoslices of original data. Observing the nuclei cut by planes it is possible to see the white contour line of original data. Figure 8 shows instead the segmentation of first and last volume of our dataset. Let us observe that during the acquisition the number of cells strongly increases. As previously introduced, this number can be quantified by counting the connected components extracted from the segmented surfaces. We show in Figure 9 an example of connected components extraction on last volume of the time lapse series. The number of nuclei detected in our dataset during the embryo development is instead plotted as graph in Figure 10. At the beginning we detected 333 nuclei, whereas at the end 520. The most parts of cells divide during the course of images acquisition. Nevertheless we would like to point out that the number of detected cells is not strictly increasing but shows small oscillations around an exponential trend. This behavior occurs because the cells are moving and the imaging includes only the top part of the embryo. Cells close to the bottom of the imaged volume can then be located either on the inside or on the outside.

In order to estimate the ability of our method of correctly extracting the number of cells, we then compared the number of nuclei identified by a manual computation on the first volume of the time lapse series (340 nuclei) with the number automatically detected. By our evaluation the correct cells number is then slightly underestimated, but our error is less than 3%. For the most part, nuclei are correctly detected and segmented. The main source of error is given by nuclei very close, if not overlapped, in original data. In this case we segment only one region. This drawback could be overcome by combining signal of both nuclei and membranes, by refining the segmentation locally, or through the multiphase level-set approach [26].

## 5. Conclusions

We applied the active contour model without edges to time lapse series of confocal images representing cells nuclei in a live zebrafish embryo. As the nuclei in our dataset are varying in shape and size, we considered two forms of the originally proposed model equation. The two formulations are based on a different approximation of the Dirac function. We then compared their performances in segmenting objects of different forms and dimensions. The maximum and total errors have been quantified as the Hausdorff distance and the mean Hausdorff distance between the data and a gold standard obtained through manual segmentation. This analysis has been made on both synthetic and real data. We proved that our suggestion of using *δ* = 1 is the formulation with the best performances. This form of the active contour model without edges has then be applied to a dataset which represents the development of a zebrafish embryo between the shield and the sphere stage. From the results of segmentation have been afterwards extracted the connected components, whose counting allowed to quantify the number of cells. The corresponding error has been estimated through a comparison between the number of cells detected by the algorithm and the number of cells detected by a manual computation in a selected volume.

## Figures and Tables

**Table 1:** The mean Hausdorff distance (MHD) and the Hausdorff distance (HD) (in *μ*m) between a gold standard of real data and the segmented surfaces using (5). HD and MHD are averaged on the number of nuclei in the subvolume. The segmentation is performed using different values of *μ*.

*μ*	MHD	HD
0.05	0.30 ± 0.10	1.42
0.1	0.35 ± 0.15	1.54
0.15	0.52 ± 0.20	1.77
